# Exhaled Breath Analysis for Lung Cancer Detection: From VOCs Signatures to EBC‐Based Liquid Biopsy and Clinical Translation

**DOI:** 10.1111/1759-7714.70404

**Published:** 2026-10-19

**Authors:** Penghai Sun, Zhenkai Fu, Shaodong Wang, Yun Li, Mantang Qiu

**Affiliations:** ^1^ Department of Thoracic Surgery Peking University People's Hospital Beijing China; ^2^ Thoracic Oncology Institute Peking University People's Hospital Beijing China; ^3^ Research Unit of Intelligence Diagnosis and Treatment in Early Non‐Small Cell Lung Cancer Chinese Academy of Medical Sciences, 2021RU002 Beijing China; ^4^ Institute of Advanced Clinical Medicine Peking University Beijing China; ^5^ Frontiers Science Center for Cancer Integrative Omics Peking University People's Hospital Beijing China

**Keywords:** biomarkers, exhaled breath analysis, exhaled breath condensate (EBC), lung cancer, volatile organic compounds (VOCs)

## Abstract

Lung cancer remains the leading cause of cancer‐related mortality globally. Although low‐dose computed tomography (LDCT) is the current standard for early screening, its high false‐positive rates and cumulative radiation exposure create a pressing clinical need for complementary, non‐invasive diagnostic tools. Exhaled breath analysis represents an accessible strategy for early detection and risk stratification. Early breathomics primarily focused on gas‐phase volatile organic compounds (VOCs). However, as volatile metabolites, VOCs are chemically unstable and highly susceptible to background ambient air and physiological confounders. Distinct from gas‐phase analysis, exhaled breath condensate (EBC) offers enhanced biomarker stability and functions as a lung‐proximal liquid biopsy. By capturing non‐volatile, high‐molecular‐weight analytes, including tumor‐derived nucleic acids and proteins, EBC more closely reflects the underlying molecular mechanisms of the respiratory microenvironment. Nevertheless, the clinical translation of EBC remains constrained by severe sample dilution, unstandardized collection device architectures, and pre‐analytical inconsistencies. This review summarizes the progression from VOCs profiling to EBC‐based platforms, detailing recent advances in sample harvesting, ultrasensitive detection technologies, and multi‐omics integration. Advancing EBC toward routine clinical use requires consensus‐driven standard operating procedures and the application of machine learning algorithms to high‐dimensional datasets. Future efforts should prioritize large‐scale, multicenter prospective trials to validate specific EBC signatures, thereby establishing this technology as a reliable liquid biopsy for precision pulmonary oncology.

## Graphical abstract

This review outlines the comprehensive workflow in exhaled breath analysis utilizing both VOCs and EBC. We detail the specific processes from sample collection and processing to distinct biomarker categorization and advanced detection technologies. Integrating these dual approaches with AI‐driven data analysis provides a powerful non‐invasive tool for early lung cancer detection.

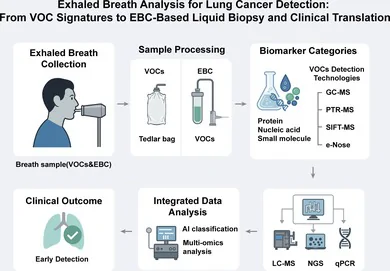

## 1 Introduction

Globally, lung cancer persists as the leading cause of cancer‐related mortality, responsible for 1.8 million deaths annually (representing 18% of total cancer deaths), primarily attributable to late‐stage diagnoses occurring in approximately 70% of patients, which severely restricts treatment options and contributes to low survival rates [1]. Current screening methods, particularly low‐dose computed tomography (LDCT), have demonstrated efficacy in early detection but suffer from notable limitations—including high false‐positive rates (as high as 96.4% in certain cohorts), cumulative radiation exposure, and restricted availability in resource‐limited regions [2, 3]. In clinical practice, these challenges translate into repeated imaging, invasive diagnostic procedures, patient anxiety, and increased healthcare costs [4]. Therefore, these constraints underscore the urgent need for non‐invasive and widely accessible diagnostic tools that can refine risk stratification, assist in the evaluation of LDCT‐detected nodules, and support longitudinal monitoring.

Exhaled breath analysis has emerged as a promising frontier in this context, offering a safe, non‐invasive, and potentially cost‐effective method for real‐time disease monitoring. Biomarkers in exhaled breath are typically divided into two primary categories: volatile organic compounds (VOCs) and components of exhaled breath condensate (EBC) (Table 1). VOCs are low‐molecular‐weight compounds that have been linked to oxidative stress, lipid peroxidation, altered energy metabolism, and tumor‐associated host responses. Multiple studies have reported VOCs panels capable of distinguishing lung cancer patients from healthy or disease controls with encouraging diagnostic performance [5].

**TABLE 1:** Comparative characteristics, advantages, and limitations of volatile organic compounds (VOCs) and exhaled breath condensate (EBC) analysis in lung cancer.

Comparison domain	Volatile organic compounds (VOCs)		Exhaled breath condensate (EBC)	
	Advantages	Limitations	Advantages	Limitations
Sample and analytes	Gas‐phase metabolites, including aldehydes, ketones, alkanes, alcohols, and aromatics; sampling is rapid, non‐invasive, and repeatable.	Highly susceptible to ambient air, humidity, smoking, diet, and other exogenous or physiological influences.	Condensed airway lining fluid containing DNA/RNA, miRNAs, extracellular vesicles, proteins, cytokines, lipids, and soluble metabolites.	Samples are highly diluted, and analyte yield varies with the collection device and sampling conditions.
Biological readout	Captures a metabolic breathprint related to oxidative stress, lipid peroxidation, energy metabolism, and host‐microbiome interactions.	Signals are indirect and generally have limited tumor specificity.	Provides lung‐proximal molecular signals, including driver mutations, methylation, miRNA dysregulation, inflammatory mediators, and tumor‐microenvironment features.	Biological interpretation is affected by variable dilution and uncertainty regarding the airway or aerosol origin of analytes.
Analytical platforms	GC–MS, PTR‐MS, SIFT‐MS, IMS, HPPI‐TOFMS, and e‐nose platforms; workflows are relatively mature and rapid screening is feasible.	Pattern‐based e‐nose systems provide limited molecular annotation; performance is affected by calibration, device drift, and algorithm transferability.	LC–MS/MS, NMR, ELISA, ddPCR, NGS, extracellular‐vesicle isolation, and nanobiosensors; compatible with molecular profiling and multi‐omics integration.	Complex preprocessing, enrichment, and ultrasensitive detection are often required.
Clinical positioning	Applicable to high‐risk screening, LDCT‐adjacent triage, benign‐malignant nodule discrimination, and longitudinal breathprint monitoring.	Not yet suitable as a stand‐alone diagnostic; most models still require prospective external validation.	Potential applications include LDCT‐positive nodule stratification, molecular subtyping, treatment‐response assessment, resistance monitoring, and postoperative surveillance.	Clinical evidence remains limited and is concentrated mainly in small, early translational studies.
Evidence base and standardization	Larger evidence base than EBC, with increasingly mature sampling and analytical workflows and growing external validation.	Sampling fraction, background correction, humidity, device drift, and model transferability remain important sources of variability.	The ERS technical framework provides a basis for standardized collection and reporting.	Consensus is still lacking for dilution correction, saliva contamination control, storage stability, freeze–thaw effects, normalization, and reference materials.
Translational outlook	A near‐term adjunct for rapid, non‐invasive screening and optimization of the LDCT pathway.	Requires locked diagnostic models and prospective multicenter validation before routine implementation.	A lung‐proximal liquid biopsy with potential for precision monitoring and integrated molecular profiling.	Requires standardized collection, analyte enrichment, molecular‐assay validation, and scenario‐specific clinical trials.

Abbreviations: ddPCR, droplet digital polymerase chain reaction; EBC, exhaled breath condensate; ERS, European Respiratory Society; GC–MS, gas chromatography–mass spectrometry; HPPI‐TOFMS, high‐pressure photon ionization time‐of‐flight mass spectrometry; IMS, ion mobility spectrometry; LC–MS/MS, liquid chromatography–tandem mass spectrometry; LDCT, low‐dose computed tomography; NGS, next‐generation sequencing; NMR, nuclear magnetic resonance; PTR‐MS, proton‐transfer‐reaction mass spectrometry; qPCR, quantitative polymerase chain reaction; SIFT‐MS, selected‐ion‐flow‐tube mass spectrometry; VOCs, volatile organic compounds.

Analytical technologies such as gas chromatography–mass spectrometry (GC–MS), electronic noses for pattern recognition, ion mobility spectrometry for rapid detection, and surface‐enhanced Raman scattering for ultrasensitive molecular analysis have further advanced VOCs‐based breathomics [6, 7, 8]. Nevertheless, VOCs signatures are inherently sensitive to smoking, diet, medication, comorbid respiratory disease, ambient air, humidity, and device‐related variability [9]. Moreover, no single VOCs have shown sufficient specificity for lung cancer, and many high‐performing models remain limited by small cohorts, case–control designs, and insufficient external validation [10, 11].

These limitations have shifted increasing attention from breath‐based pattern recognition alone toward biomarkers that may more directly capture tumor‐associated molecular events. EBC provides such an opportunity by collecting condensed exhaled water vapor and aerosolized droplets derived from the airway lining fluid. Unlike VOCs profiling, which primarily reflects volatile metabolic signatures, EBC enables analysis of non‐volatile and particle‐associated biomarkers, including DNA, RNA, microRNAs, extracellular vesicles, proteins, cytokines, lipids, and water‐soluble metabolites [12]. This broader molecular spectrum positions EBC as a lung‐proximal liquid biopsy that may bridge breathomics with precision oncology. Importantly, EBC‐based studies have reported the detection of oncogenic mutations, resistance‐associated alterations, methylation changes, deregulated microRNAs, inflammatory mediators, and oxidative stress markers in patients with lung cancer [13, 14, 15]. These findings suggest that EBC may provide information beyond diagnosis, including molecular subtyping, treatment response assessment, resistance monitoring, and postoperative surveillance. Furthermore, integrating breathomics with multi‐omics approaches, particularly those targeting cancer‐related genetic and epigenetic alterations, holds promise for improving biomarker specificity and clinical utility [2, 16].

Despite its biological appeal, EBC analysis remains less mature than VOCs profiling and faces substantial methodological barriers. EBC samples are highly diluted, analyte concentrations are often low, and results are influenced by condensation temperature, breathing pattern, collection device, saliva contamination, storage conditions, freeze–thaw cycles, and normalization strategy. These pre‐analytical variables have limited reproducibility and hindered comparison across studies. Recent advances in standardized collection protocols, microfluidic enrichment, nanomaterial‐enhanced sensing, ultrasensitive PCR and sequencing, extracellular vesicle isolation, and wearable EBC harvesting devices may help overcome these barriers and accelerate clinical translation [12, 15, 16]. Accordingly, this review summarizes recent progress in exhaled breath analysis for lung cancer, with VOCs profiling discussed as the more established breathomics framework and EBC emphasized as an emerging molecular platform with particular potential for lung‐proximal liquid biopsy, LDCT‐adjacent risk stratification, and longitudinal disease monitoring.

## 2 Volatile Organic Compounds Analysis

### 2.1 Biomarkers and Metabolic Relevance of VOCs

The biological origins of VOCs in lung cancer are primarily attributed to altered metabolic pathways resulting from carcinogenesis. Exhaled VOCs originate from both endogenous metabolic processes and exogenous sources (e.g., environmental exposures or smoking), with endogenous VOCs serving as critical biomarkers for disease pathophysiology [17, 18].

Lung cancer cells undergo significant metabolic reprogramming that alters VOCs production through multiple interconnected pathways. For instance, elevated oxidative stress within the tumor microenvironment induces lipid peroxidation, degrading polyunsaturated fatty acids to generate specific alkanes (e.g., pentane, decane) and saturated aldehydes (e.g., hexanal, pentanal, octanal, and nonanal) [5, 18, 19]. Demonstrating the diagnostic utility of these specific biomarkers, Sun et al. utilized proton transfer reaction‐mass spectrometry (PTR‐MS) coupled with linear discriminant analysis to successfully distinguish lung cancer patients from healthy cohorts based on the discrete ion signals of these aldehydes, alongside ketones and alcohols [20]. As a hallmark of cancer metabolism characterized by increased aerobic glycolysis and impaired mitochondrial oxidative phosphorylation, the Warburg effect drives the production of ketones (e.g., acetone, 2‐butanone) and short‐chain alcohols (e.g., 1‐propanol, ethanol, and 1‐butanol) [18, 21]. Notably, 3‐hydroxy‐2‐butanone, a byproduct of pyruvate metabolism, is consistently enriched in the breath of non‐small cell lung cancer (NSCLC) cohorts [22, 23]. Furthermore, disruptions in amino acid metabolism, particularly within the phenylalanine and tryptophan pathways, can yield aromatic hydrocarbons such as styrene and nitrogen‐containing compounds like acetonitrile, potentially mediated by microbial‐host interactions [24, 25, 26].

Despite the mechanistic plausibility of these specific VOCs as cancer biomarkers, their clinical translation is hindered by several critical challenges. A major bottleneck is the extreme susceptibility of VOCs profiles to a broad spectrum of confounding factors. Endogenously, breath VOCs profiles are frequently confounded by diverse multi‐system disorders, ranging from chronic respiratory diseases (such as COPD and asthma) to broader systemic conditions [27]. Exogenously, these profiles are inherently sensitive to smoking status, dietary intake, medication history, and various environmental or occupational exposures. Furthermore, the pathophysiological complexity of tumor biology, characterized by spatial and temporal heterogeneity, complicates inter‐study reproducibility [17]. These biological variations are exacerbated by methodological inconsistencies, including differences in breath sampling techniques, sample storage, and analytical platforms. Therefore, while specific exhaled VOCs mirror lung cancer‐related metabolic shifts, rigorous validation in large, stringently matched cohorts and methodological harmonization are essential to advance them toward reliable clinical biomarkers.

### 2.2 Core Collection Technologies of VOCs

Standardized sample collection and storage protocols are critical for the reliability of exhaled volatile organic compound analysis. The optimal sampling strategy depends on the clinical setting and the specific analytical platform, such as gas chromatography–ion mobility spectrometry. Current approaches include real‐time online analysis, where patients exhale directly into the device, and offline sampling, which is necessary when point‐of‐care analysis is unfeasible. In offline workflows, Haldane tubes combined with disposable syringes allow for the targeted collection of alveolar air, frequently referred to as end‐tidal breath. This method excludes the initial respiratory dead‐space volume. Because alveolar air originates directly from alveolar blood‐gas exchange, it accurately reflects systemic metabolism; however, these samples require immediate instrumental injection. When off‐site transport or delayed analysis is required, polymer bags, most commonly Tedlar bags, are frequently used to collect mixed expiratory breath due to their chemical inertness, durability, and cost‐effectiveness. This fraction contains both alveolar and dead‐space air, inherently introducing confounding volatiles from the oro‐nasal cavities, upper gastrointestinal tract, and ambient environment. Alternative containers like gas‐tight syringes and glass bulbs are limited by volume and fragility, whereas passivated stainless‐steel canisters are bulky and expensive despite their superior stability. Furthermore, storing samples in polymer bags introduces significant methodological limitations. The original volatile profile can be altered by transmembrane gas diffusion, external volatile penetration, wall adsorption, polymer off‐gassing, and chemical reactions among reactive compounds. Therefore, storage time fundamentally dictates sample integrity. While standard gas mixtures may remain stable in Tedlar bags for extended periods, authentic breath samples degrade rapidly. To minimize sample distortion and ensure clinical validity, analysis should be completed within 1 h of ambient collection, maintaining a recovery rate exceeding 80% for sensitive targets like carbonyl compounds.

### 2.3 Key Detection Technologies for VOCs Profiling

The detection of exhaled VOCs in lung cancer relies on highly sensitive and selective analytical platforms, which can be broadly categorized into chromatography‐mass spectrometry (MS)‐based techniques, direct MS methods, and sensor‐based systems. GC–MS remains the gold standard for VOCs identification and quantification [11]. It offers high resolution and sensitivity, especially when coupled with solid‐phase microextraction (SPME) for pre‐concentration, allowing detection at parts‐per‐billion (ppb) levels. Lung cancer‐specific VOCs such as 1‐butanol and 3‐hydroxy‐2‐butanone have been reliably detected using this technique [19]. Two‐dimensional GC (GC × GC) further enhances separation capability for complex breath samples, as demonstrated in studies such as Ma et al., which identified potential biomarkers including acetone, isoprene, and propanol for NSCLC detection [8, 28]. However, Sakumura et al. showed that a combination of VOCs, such as isoprene and propanol, can achieve approximately 89% accuracy in lung cancer screening [29]. GC‐based methods are time‐consuming, often requiring over 30 min per sample, and are susceptible to artifact formation due to chemical interactions with adsorbents, as highlighted in multiple studies [30].

To overcome limitations in speed and portability, direct MS approaches have gained traction. Selected Ion Flow Tube‐MS (SIFT‐MS), an real‐time technique, quantifies trace breath VOCs such as acetaldehyde in a matter of seconds, as demonstrated in studies on lung cancer cells [20]. Proton Transfer Reaction‐MS (PTR‐MS) enables real‐time VOCs detection without the need for sample pre‐concentration. Sun et al. used PTR‐MS to distinguish lung cancer patients by analyzing ion signals corresponding to aldehydes, ketones, and alcohols (e.g., m/z 45 for acetaldehyde, m/z 59 for acetone, and m/z 43/61 for propanols) through linear discriminant analysis [28]. Emerging direct MS platforms address humidity interference—a major limitation in breath analysis. High‐pressure photon ionization time‐of‐flight mass spectrometry (HPPI‐TOFMS) demonstrates exceptional tolerance to water vapor while maintaining high sensitivity without sample pretreatment. In a blinded validation study of 428 participants, HPPI‐TOFMS combined with Support Vector Machines achieved 100% sensitivity and 92.86% specificity, outperforming conventional MS methods in accuracy (AUC = 0.9586). Its rapid analysis (< 60 s/sample) and standardized alveolar air collection (via CO_2_‐sensor‐controlled sampling) highlight clinical utility for real‐time screening [31]. These platforms provide rapid, in situ analysis but require substantial expertise and equipment calibration, particularly for distinguishing low‐abundance VOCs from background air contaminants.

Sensor‐based systems, particularly electronic noses (eNoses), represent a practical alternative for point‐of‐care applications [25]. These devices utilize cross‐reactive sensor arrays—such as metal oxide semiconductors or conductive polymers—to detect VOCs patterns without identifying individual compounds. For instance, Chen et al. reported 93.59% accuracy in distinguishing lung cancer patients from healthy controls using MOS‐based eNoses pattern recognition [32]. Additional techniques such as needle trap devices (NTDs) are emerging as effective pre‐concentration tools for VOCs analysis in breath samples, enabling sensitive detection of disease biomarkers when coupled with laboratory‐based GC–MS [26]. In comparative terms, GC–MS provides the highest specificity, while eNoses and surface‐enhanced Raman scattering offer rapid, portable alternatives. The future of lung cancer VOCs diagnostics will likely involve hybrid strategies combining sensitivity, speed, and usability across clinical contexts.

## 3 Exhaled Breath Condensate Analysis

### 3.1 EBC Components and Their Biological Association With Lung Cancer

Exhaled Breath Condensate, a non‐invasively collected biofluid derived from airway lining fluid, captures both volatile and non‐volatile molecules that reflect underlying pulmonary pathophysiology. Its complex composition—including nucleic acids, proteins, and metabolites—offers valuable insights into the molecular alterations associated with lung cancer, supporting its potential in early detection, disease monitoring, and treatment stratification.

Genetic and epigenetic alterations in EBC closely mirror intratumoral changes. Mutations in key oncogenes (e.g., KRAS, EGFR) and tumor suppressors (e.g., TP53, p16) have been identified in EBC samples, with KRAS mutations showing strong concordance with tumor tissue and dynamic changes following surgical resection [33]. Similarly, resistance‐associated EGFR T790M mutations have been detected in EBC, highlighting its role in therapeutic monitoring [34]. Aberrant methylation of genes such as p16 in EBC demonstrates high specificity for NSCLC [35]. Furthermore, deregulated microRNAs—particularly miR‐21, and let‐7—are commonly enriched in EBC, with elevated miR‐21 correlating with tumor progression and poor prognosis, and reduced let‐7 levels reflecting its tumor‐suppressive function [36, 37].

Proteomic profiling of EBC has uncovered cancer‐related proteins involved in inflammation, oxidative stress, and immune modulation. For instance, overexpression of dermcidin and S100A9, both linked to tumor proliferation and metastasis, distinguishes lung cancer from healthy states and correlates with disease aggressivenes [38]. Pro‐inflammatory cytokines such as IL‐1β and VEGF reflect the tumor‐associated inflammatory milieu and angiogenic processes [39, 40]. Adhesion molecules like sICAM‐1 and sVCAM‐1 are elevated in NSCLC patients and correlate with tumor‐associated inflammation [41]. Surfactant proteins (SP‐A, SP‐D), often altered in lung cancer patients with COPD, further support the use of EBC in multi‐marker panels [42]. EBC metabolomic profiling reveals distinct patterns associated with cancer metabolism, particularly markers of oxidative stress such as elevated 8‐isoprostane [14, 43]. Lipid alterations, including monopalmitin and squalene, are observed in lung cancer patients [44].

Due to the low concentration of analytes in EBC, stringent standardization in sample collection and storage and advanced detection platforms (e.g., PCR, NGS) are essential. Despite these challenges, EBC provides real‐time insights into tumor biology—capturing mutations and disease‐specific biomarkers—positioning it as a promising non‐invasive “liquid biopsy” [45, 46].

### 3.2 Collection Devices of EBC

EBC collection enables non‐invasive detection of pulmonary diseases by capturing aerosolized components in exhaled breath, providing a safer alternative to conventional invasive procedures while allowing for longitudinal monitoring. In recent years, EBC collection devices have evolved from simple passive cooling systems, exemplified by the R‐Tube, to more advanced active sampling platforms that integrate dynamic temperature control and inertial impaction mechanisms, demonstrating advantages in improving sampling efficiency and reproducibility.

Conventional EBC collection devices are primarily based on low‐temperature condensation, whereby a cooled interface facilitates the conversion of exhaled water vapor and aerosols into liquid samples, enabling the enrichment of both volatile and non‐volatile biomarkers. Commonly used platforms include EcoScreen, TurboDECCS, and R‐Tube, which differ in temperature control mechanisms, device size, and clinical applicability. EcoScreen operates under stable low‐temperature conditions (< −15°C), offering high biomarker recovery and reproducibility, making it well‐suited for research settings and longitudinal studies [47]. However, it is limited by its bulky design, high cost, and operational complexity. TurboDECCS employs electronically controlled cooling to achieve improved temperature stability and can be integrated with mechanical ventilation systems, rendering it suitable for critically ill patients and continuous monitoring [48]. In contrast, R‐Tube is lightweight and cost‐effective, with high collection efficiency over a short period, making it more applicable for routine clinical screening and primary care settings, although it exhibits limited cooling stability [49]. Overall, conventional EBC collection systems typically require 10–15 min to obtain approximately 1 mL of condensate, which constrains their utility for real‐time monitoring. Furthermore, these approaches continue to face challenges, including lack of standardized protocols, uncertainty regarding aerosol sources, and limited detection sensitivity for low‐abundance biomarkers [50].

In recent years, novel EBC collection strategies based on dynamic temperature‐controlled collection techniques and inertial impact have emerged to address the limitations of conventional condensation methods, particularly with respect to sample dilution and compositional selectivity. By integrating real‐time respiratory parameters such as tidal volume, exhaled breath temperature, and carbon dioxide concentration, dynamic temperature‐controlled sampling enables effective differentiation of alveolar air from anatomical dead space [51]. However, these systems typically require the integration of multiple sensors and microcontroller units, resulting in increased system complexity, higher cost, and limited portability. Consequently, current applications are largely confined to mechanistic studies and high‐precision metabolic monitoring. In contrast, inertial impact‐based sampling exploits the aerodynamic properties of aerosol particles. By inducing abrupt directional changes in airflow through multi‐stage microstructures, particles with larger aerodynamic diameters (typically > 300 nm) are selectively captured based on their inertia. This approach does not require low‐temperature condensation, thereby minimizing the impact of thermal fluctuations on sample integrity and enabling efficient preservation of nucleic acids (RNA/DNA) within non‐volatile biological particles such as viruses and bacteria, with reported capture efficiencies exceeding 85% [52]. Nevertheless, its capability to enrich volatile small molecules (e.g., VOCs) is limited, and the reliance on precision microfabrication increases technical complexity and cost. Therefore, while inertial impaction is well‐suited for pathogen‐focused applications, its utility in metabolomics and small‐molecule biomarker analysis remains constrained.

Different from the above three collection methods, wearable smart masks (e.g., EBCare) integrating passive cooling, microfluidic channels, and biosensing components have enabled continuous sample collection with wireless analytical readout [16]. Such innovations enhance temporal resolution and patient compliance, although challenges related to operational stability, user comfort, and production cost remain.

### 3.3 Core Detection Technologies of EBC

EBC biomarker detection relies on sensitive technologies tailored to the low‐abundance nature of its constituents. These include nanomaterial‐enhanced sensors, molecular diagnostic techniques, and integrated multi‐omics platforms.

Nanoparticles significantly enhance the sensitivity of EBC biomarker detection. Gold nanoparticles (AuNPs) enable field‐amplified sample stacking (FASS) for polyamine separation, achieving nanogram‐level detection limits (0.07–0.17 ng/mL) [53]. Copper nanoparticle/polyaniline composites facilitate rapid electrochemical detection of H_2_O_2_ at picomolar levels (LOD: 0.0026 nM) [54]. Meanwhile, β‐cyclodextrin‐modified AuNPs improve small‐molecule detection via Rayleigh scattering, with a detection limit of 0.003 μg/mL for phenytoin [55]. Surface‐enhanced electrochemiluminescence (SEECL) platforms further allow multiplexed immunodetection of cancer antigens at picogram‐level sensitivity, outperforming conventional ELISA [56].

Mass spectrometry (LC–MS/MS) enables proteomic profiling of EBC samples, as demonstrated in studies analyzing post‐spaceflight adaptations [57]. Detection of oncogenic mutations (e.g., EGFR T790M, KRAS) in EBC is feasible using ultrasensitive PCR‐based platforms like UltraSEEK combined with NGS validation [58]. Additionally, RT‐qPCR quantification of miR‐21 in EBC shows consistent upregulation in NSCLC patients, supporting its diagnostic utility [36, 59].

Multi‐omics platforms combine proteomic, metabolomic, and genomic data for comprehensive disease profiling. Integration of these omics layers enables comprehensive disease mechanism analysis. For instance, multi‐omics frameworks combine inflammatory proteins (e.g., TNF‐α), metabolites (e.g., NH_4_^+^), and genomic alterations to model respiratory and systemic disorders, enhancing early screening and personalized therapeutic monitoring [16].

Despite technological advances, EBC analysis remains challenged by sample variability, extreme dilution/sample dilution, and concentration artifacts, and device heterogeneity [60, 61]. Future strategies should prioritize standardization, microfluidic integration, and wearable sensors to enable point‐of‐care diagnostics.

### 3.4 Clinical Value of Biomarkers

The clinical application of EBC biomarkers spans the entire continuum of lung cancer management. Capitalizing on the noninvasive nature of breath testing, these molecular signatures exhibit substantial translational potential and can be categorized into three primary clinical scenarios: the early screening of high‐risk populations, the longitudinal monitoring of tumor progression, and the evaluation of therapeutic drug resistance.

#### 3.4.1 Early Screening in High‐Risk Populations

As a biofluid collected directly from the airway and alveolar surfaces, EBC more authentically reflects the local pulmonary microenvironment compared to conventional imaging modalities such as LDCT. Consequently, specific molecular biomarkers within EBC, encompassing oxidative stress and inflammatory indicators, DNA methylation profiles, microRNAs (miRNAs), metabolites, and proteomic signatures, demonstrate significant potential value for the early diagnosis of lung cancer.

Specifically, the evaluation of oxidative stress and inflammatory biomarkers in EBC provides a reliable approach for the screening for high‐risk populations of lung cancer. In 2009, Chan et al. demonstrated that EBC from patients with NSCLC presents significantly elevated H_2_O_2_ levels and diminished antioxidant capacity compared to smokers and non‐smokers, positioning oxidative stress indices as promising candidates for early screening [62]. A study conducted by Dalaveris et al. in the same year revealed that TNF‐α levels can effectively differentiate patients with NSCLC from those with COPD [63]. Furthermore, the detection of 8‐isoprostane, a classic marker of lipid peroxidation, has exhibited extraordinary diagnostic precision for lung cancer screening, achieving a peak sensitivity of 100% alongside a specificity of 99.2% [50]. At the epigenetic and transcriptomic levels, hypermethylation of the RASSF1A and APC promoters in EBC achieves a screening sensitivity of 70%–85% for NSCLC [34], whereas p16 methylation, despite its high specificity, is limited by a low detection rate of approximately 40% [35]. Furthermore, miRNA panels, notably involving the let‐7 family, demonstrate robust diagnostic efficacy in early differentiation; for instance, EBC let‐7i yields an area under the curve (AUC) of 0.955, with 75% sensitivity and 95% specificity [44], while combinatorial panels (e.g., miR‐4507, miR‐6777‐5p, and miR‐451a) can further refine early detection capabilities [64]. Metabolomic profiling also reveals that specific lipid alterations (e.g., monopalmitin) mirror early localized metabolic reprogramming in the lung, serving as potential auxiliary screening tools [44]. From a proteomic perspective, an LC–MS/MS analysis of EBC conducted by López‐Sánchez et al. identified 348 proteins across various cohorts, with 288 detected in lung cancer patients. The lung cancer cohort exhibited a significantly higher mean protein count, and establishing a diagnostic threshold of 12 or more identified proteins yielded an AUC of 0.8, alongside 70% sensitivity and 67% specificity. Notably, increased EBC detection rates of the hemoglobin beta subunit and distinct histone subtypes (H2A, H2B, and H4) in lung cancer, consistent with alveolar epithelial hemoglobin expression, underscore their diagnostic relevance in pulmonary physiopathology and early oncological transitions [65].

#### 3.4.2 Longitudinal Monitoring of Tumor Progression

EBC biomarkers offer utility beyond the risk stratification of pulmonary nodules, playing a pivotal role in the longitudinal monitoring of lung cancer patients. Specifically, these biomarkers demonstrate substantial potential in evaluating clinical staging, assessing tumor burden, and tracking prognosis during post‐treatment follow‐up.

Regarding pathological classification and metastasis prediction, prior evidence indicates that aberrant elevations of S100A9 and dermcidin in EBC are closely associated with invasive phenotypes and distant metastasis in lung adenocarcinoma, effectively reflecting aggressive tumor biology [38]. Furthermore, inflammatory and angiogenic factors in EBC closely mirror clinical staging; for instance, the expression levels of IL‐1β and VEGF correlate significantly with the TNM staging of NSCLC [39]. In the context of tumor burden assessment, proteomic investigations have revealed that the concentrations of most cytokeratins exhibit a robust positive correlation with tumor volume. This correlation is particularly pronounced for KRT1, KRT9, KRT10, and KRT6C, thereby providing direct molecular insights into the real‐time growth status of the tumor in vivo. Finally, for prognosis tracking and dynamic follow‐up, advancements in analytical technologies have expanded the scope of EBC monitoring from conventional proteins to dynamic alterations in nucleic acids at the epigenetic level. In 2023, Aslı Tetik Vardarlı et al. longitudinally analyzed EBC samples from 40 patients with Stage III to IV lung adenocarcinoma at baseline and 3 months post‐treatment. Their evaluation of six tumor‐associated long non‐coding RNAs (lncRNAs) revealed that the fold changes of these transcripts exhibited marked dynamic fluctuations following therapeutic intervention [60]. Although constrained by a short follow‐up period that precluded direct correlation with long‐term survival, these prominent variations strongly highlight the clinical potential of EBC lncRNAs for non‐invasively tracking tumor evolution.

#### 3.4.3 Evaluation of Therapeutic Drug Resistance

Nucleic acid EBC biomarkers, notably circulating tumor DNA (ctDNA), offer distinct diagnostic advantages for resistance evaluation. The excessively high background of wild‐type nucleic acids and potent endogenous nuclease activity in the blood significantly limit the detection sensitivity for trace resistance‐associated mutant genes. As acellular, organ‐specific fluid, EBC features a negligible wild‐type nucleic acid background and substantially lower nuclease activity than the systemic circulation, conferring a higher theoretical sensitivity for capturing resistance‐associated ctDNA mutation signals [61]. Smyth et al. demonstrated in 2018 that EBC samples yielded a significantly higher detection rate of the critical EGFR T790M resistance mutation compared to plasma in advanced lung cancer cohorts [66]. This diagnostic superiority was particularly pronounced when patients exhibited intrathoracic disease progression within the lung parenchyma or mediastinal lymph nodes. These findings indicate that monitoring resistance‐associated ctDNA via EBC can effectively compensate for the inherent limitations of conventional plasma testing.

### 3.5 Limitations and Optimization Directions

EBC analysis faces significant reproducibility challenges due to dilution variability, sample contamination, and inconsistent protocols, which stem from the inherent complexities of sample collection and processing [14, 67]. Although EBC offers a noninvasive route for tumor biomarker discovery, these limitations impede its broader clinical adoption. Translating findings into reliable clinical tools requires large‐scale validation and the integration of multi‐omics data to enhance diagnostic accuracy and utility [68].

Key limitations include dilution and standardization issues, as EBC consists of over 99% water, complicating quantification and leading to inter‐study variability without robust normalization strategies such as conductivity or urea correction [69]. Additionally, the trace concentrations of biomarkers necessitate ultra‐sensitive detection methods that surpass conventional techniques like ELISA or PCR, posing analytical challenges [70]. Pre‐analytical and technical hurdles, such as risks of saliva or air contamination, degradation from improper storage, and matrix effects that reduce sensor accuracy, further compromise reliability and reproducibility [71].

Optimization strategies focus on protocol standardization through ERS‐recommended guidelines for collection and correction, ensuring consistency across studies [15]. Advanced technologies, including nanomaterial‐enhanced sensors (e.g., AuNPs for H_2_O_2_ detection), microfluidic platforms, and high‐throughput omics approaches, improve sensitivity and enable multi‐analyte profiling [13, 72]. Rigorous biobanking practices with strict storage conditions and cohort‐scale biomarker validation are also vital to address variability and enhance clinical relevance.

Through these methodological refinements and integrative analyses, EBC‐based diagnostics are poised to complement existing tools, facilitating early detection and dynamic management of lung cancer while advancing personalized medicine approaches.

## 4 Discussion

### 4.1 VOCs Versus EBC: Technical Comparison

VOCs are gaseous metabolites captured directly from exhaled breath using polymer bags, canisters, or thermal desorption tubes. Analytical platforms such as GC–MS, e‐noses, and sensor arrays are employed for their identification and quantification [2, 34, 73]. In contrast, EBC collects non‐volatile constituents—including proteins, nucleic acids, and metabolites—by condensing exhaled air onto a cooled surface, forming an aqueous matrix that is typically analyzed using LC–MS/MS, immunoassays, or PCR‐based techniques [12, 13]. While both techniques are non‐invasive and amenable to repeated sampling, EBC presents greater technical challenges due to its high‐water content (> 99%), requiring pre‐analytical concentration via lyophilization, ultrafiltration, or solid‐phase extraction [12, 74]. A comprehensive overview of the major analytical platforms employed in exhaled breath analysis is provided in Table 1, offering a side‐by‐side comparison that underscores the trade‐offs between sensitivity, speed, and clinical feasibility.

VOCs primarily reflect systemic and local metabolic alterations associated with tumorigenesis. More than 3000 VOCs—including isoprene, acetone, and alkanes—have been linked to oxidative stress, lipid peroxidation, and cellular metabolism in lung cancer [34]. However, their trace‐level abundance (ppb–ppt range) necessitates ultra‐sensitive detection, and their susceptibility to exogenous interference (e.g., environmental contaminants) poses a limitation [73]. Conversely, EBC enables direct assessment of respiratory tract inflammation and tumor‐related genomic alterations. Biomarkers such as H_2_O_2_, cytokines, and oncogene mutations (e.g., EGFR, TP53) detected via next‐generation sequencing provide mechanistic insights into lung carcinogenesis [34, 50, 75, 76]. However, the absence of standardized reference ranges and matrix normalization protocols hinders reproducibility and cross‐study comparability [77].

As for their advantages and Limitations, VOCs offer rapid detection (often within minutes), compatibility with portable e‐nose devices, and the capacity to distinguish histological subtypes such as NSCLC versus SCLC. However, their sensitivity to humidity, ambient air composition, and equipment costs remains practical concerns [17, 78]. EBC, in contrast, supports high‐resolution analyses of nucleic acids and protein biomarkers, enabling mutation tracking (e.g., EGFR T790M) and detection of pathogens such as *Aspergillus* [66, 79]. Yet, limitations such as complicated detection devices, low analyte concentrations, and lack of standardized sampling reduce clinical applicability [12, 74].

### 4.2 Multimodal Diagnostic Integration

The synergistic combination of VOCs and EBC biomarkers compensates for individual limitations, enhancing diagnostic precision. For instance, integrating specific VOCs (e.g., ethylbenzene, hexane) with EBC‐derived markers (e.g., H_2_O_2_) and clinical variables (age, pack‐years) significantly improved NSCLC detection (AUC: 0.80) compared to VOCs alone (AUC: 0.68). Further potential lies in coupling exhaled VOCs profiles (e.g., pentane) with EBC‐based genetic alterations (e.g., TP53 mutations) for early lung cancer screening, though rigorous clinical validation is required to establish robust sensitivity and specificity [10]. The specific biomarker classes derived from VOCs and EBC—ranging from aldehydes and ketones to microRNAs and epigenetic modifications—along with their mechanistic relevance and diagnostic performance, are summarized in Table 2, highlighting their respective contributions to non‐invasive lung cancer detection.

**TABLE 2:** Lung cancer biomarkers and their clinical significance.

Biomarker type	Representative compounds	Key advantages	Association with lung cancer	Potential mechanisms
Aldehydes (VOCs)	Hexanal, Heptanal, Nonanal, Decanal, Pentanal	Non‐invasive, rapid sampling; reflects oxidative stress and lipid peroxidation.	Significantly elevated in NSCLC (especially adenocarcinoma); diagnostic accuracy: 70%–89% (AUC 0.73–0.88).	Lipid peroxidation due to oxidative stress; tumor‐associated membrane degradation.
Ketones (VOCs)	3‐Hydroxy‐2‐butanone (Acetoin), Acetone	Distinguishes adenocarcinoma from squamous cell carcinoma; stable in breath.	Increased in adenocarcinoma (AUC 0.94–0.96); correlates with tumor histology but not stage.	Oxidative metabolism of butane; Increased oxygen free‐radical activity.
Hydrocarbons (VOCs)	Isoprene, Alkanes (C4–C10), Benzene derivatives	Detects early‐stage tumors; independent of smoking status.	Increased in lung cancer vs. controls (*p* < 0.01); MAGIIC biomarker predicts nodules (AUC 0.88).	Cytochrome P450‐mediated alkane metabolism; endogenous lipid peroxidation.
Alcohols (VOCs)	1‐Butanol, 1‐Propanol, Ethanol, Isopropanol	High sensitivity (75%–84%) for early detection; complementary to CT screening.	Increased in NSCLC (*p* < 0.001); 1‐butanol discriminates Stages I–IV (AUC 0.94).	Oxidative stress byproducts; microbial metabolism in tumor microenvironment.
Carboxylic Acids/Furan (VOCs)	Formic acid, Acetic acid, 2‐Furancarboxaldehyde	Identifies tumor subtypes; validated in perioperative dynamic breathomics.	16‐VOCs panel achieves 89% accuracy (AUC 0.95); furan derivatives linked to SCLC.	Incomplete oxidation of carbohydrates; inflammation‐driven pathways.
miRNAs (EBC)	miR‐21, miR‐155, miR‐486, let‐7, miR‐31‐3p	Tissue‐specific; non‐invasive; high diagnostic sensitivity (75%–95%).	miR‐21 Increased and miR‐486 decreased in NSCLC; miR‐155 Increased correlates with metastasis.	Oncogenic (miR‐21/miR‐155) or tumor‐suppressive (miR‐486/let‐7) regulation; modulates apoptosis and proliferation.
Proteins (EBC)	Dermcidin, S100A9, IL‐6, VEGF, TNF‐α	Reflects tumor microenvironment; quantifiable via ELISA/MS.	Dermcidin/S100A9 Increased in adenocarcinoma (*p* < 0.001); IL‐6/VEGF increased correlate with tumor stage and size.	Inflammation (IL‐6/TNF‐α); angiogenesis (VEGF); metastasis promotion (S100A9).
Metabolites (EBC)	8‐Isoprostane, H_2_O_2_, Monoacylglycerols, Squalene	Measures oxidative stress; detects high‐risk individuals.	H_2_O_2_ Increased in NSCLC (*p* < 0.001); monoacylglycerols discriminate cancer from benign nodules.	Lipid peroxidation (8‐isoprostane); mitochondrial DNA damage (H_2_O_2_).
DNA/RNA Biomarkers (EBC)	Methylated p16/RASSF1A, EGFR/KRAS mutations, lncRNAs	“Liquid biopsy” for mutations; detects early molecular changes.	p16 methylation: 40% sensitivity in EBC; ctDNA mutations concordant with tissue (82%).	Epigenetic silencing (p16/RASSF1A); driver mutations (EGFR/KRAS); metastasis regulation (lncRNAs).
Viruses/Microbiota (EBC)	HPV, EBV, Aspergillus spp.	Identifies infection‐related carcinogenesis; low‐cost screening.	HPV/EBV detected in 16%–28% of NSCLC EBC samples; fungal colonization linked to inflammation.	Viral oncogenesis (HPV E6/E7 proteins); chronic inflammation promoting tumorigenesis.

Abbreviations: AUC, area under the ROC curve; NSCLC, non‐small cell lung cancer; SCLC, small cell lung cancer.

Advanced analytical platforms amplify diagnostic capabilities. Nanotechnology‐enabled sensors, such as gold nanoparticles functionalized for VOCs detection and EBC cytokines (e.g., CEA, NSE, and SCC), achieve high specificity in subtyping lung cancer (e.g., NSCLC vs. SCLC) [71, 80]. Concurrently, machine learning algorithms (e.g., Random Forest) applied to GC–MS‐derived VOCs data demonstrate promising accuracy in discriminating benign pulmonary nodules from malignant lesions [81, 82].

Clinical translation of EBC faces significant standardization challenges. Sampling variability—including breath volume, condensation temperature, and storage protocols—compromises biomarker stability and cross‐study comparability [70]. Multi‐omics integration (e.g., genomics and metabolomics) shows promise for comprehensive disease profiling but requires rigorous methodological harmonization [50]. Prospective multicenter trials are needed to validate integrated diagnostic frameworks, such as combining E‐noses devices with EBC biomarker analysis [79].

VOCs and EBC offer complementary advantages: VOCs enable real‐time metabolic profiling, while EBC provides genomic and inflammatory insights. Their convergence, augmented by nanotechnology and AI, holds promise for precision screening. However, standardized protocols and large‐scale validation remain imperative for clinical deployment.

### 4.3 Challenges and Future Directions

Despite substantial advancements in breath‐based diagnostics for lung cancer, several critical challenges must be addressed to facilitate clinical translation and regulatory acceptance. A major barrier to reproducibility and cross‐platform validation lies in the lack of standardized protocols for breath sampling, storage, and analysis.

Differentiating tumor‐specific signals from non‐malignant sources remains a core challenge. Many VOCs and EBC biomarkers are influenced by inflammatory responses, smoking, environmental exposures, or underlying comorbidities such as asthma, COPD and diabetes [83]. This limits their diagnostic specificity, particularly in heterogeneous populations [46, 81]. Inter‐individual variability in respiratory physiology, airway microbiome composition, dietary habits (e.g., trimethylamine N‐oxide generation), and systemic metabolism significantly influences exhaled biomarker profiles. For instance, microbial byproducts and host immune responses may introduce confounding VOCs signatures unrelated to malignancy. Without robust normalization strategies, these fluctuations challenge the reproducibility and interpretability of breath‐based diagnostics [84, 85].

Moreover, low‐abundance targets—such as ctDNA or tumor‐derived miRNAs in EBC—require ultra‐sensitive detection platforms (e.g., next‐generation sequencing) that are not yet standardized for routine clinical use, as evidenced by challenges in dilution correction, inter‐study variability, and insufficient sensitivity of current assays [34, 86].

The clinical utility of breath analysis remains largely unproven in real‐world settings. Most studies to date are limited by small sample sizes, lack of longitudinal follow‐up, and geographic homogeneity. To ensure diagnostic generalizability, large‐scale, multi‐center prospective trials involving diverse cohorts—including high‐risk smokers, never‐smokers, and patients with benign lung disease—are essential [85, 87, 88]. Additionally, integrating breath diagnostics with existing modalities such as low‐dose computed tomography requires validated risk algorithms to reduce false positives and optimize patient stratification [89].

To overcome current limitations and unlock the full potential of breath omics in lung cancer, future research must prioritize several key directions: Advanced sensing technologies, including emerging platforms such as nano‐biosensors, “nose‐on‐chip” devices, and miniaturized gas chromatography/differential mobility spectrometry (GC/DMS) systems, which enable real‐time, point‐of‐care testing with enhanced sensitivity and specificity, making them particularly suitable for community‐based screening and longitudinal monitoring; Multi‐omics integration, where comprehensive diagnostic panels combine VOCs signatures with exhaled breath condensate‐derived analytes—such as miRNAs, ctDNA, proteins, and inflammatory mediators—to provide multidimensional snapshots of tumor biology, thereby enhancing diagnostic accuracy and offering mechanistic insights into lung carcinogenesis; AI‐driven diagnostics, leveraging machine learning and deep learning models to analyze high‐dimensional breath omics data, enabling complex pattern recognition that improves cancer stage and subtype classification while mitigating inter‐patient variability, with explainable AI approaches further enhancing clinician trust and regulatory transparency [79]; and crucially, establishing international guidelines for breath sample collection, biomarker quantification, quality control, and data reporting, where initiatives toward consensus protocols—akin to the MIAME standards for microarrays—are imperative to enhance reproducibility, enable meta‐analyses, and facilitate regulatory approval for clinical translation.

## 5 Conclusion

Breath analysis represents a promising non‐invasive modality for early lung cancer detection, utilizing VOCs and EBC biomarkers. While current evidence highlights its capacity to distinguish malignancies from benign pulmonary diseases, clinical translation remains hindered by a lack of methodological standardization and large‐scale biomarker validation. Integrating multi‐omics, artificial intelligence, and advanced biosensors will be critical to engineering high‐fidelity, point‐of‐care platforms. Ultimately, realizing the clinical utility of breath biopsies in precision oncology requires robust multicenter trials and synergetic integration with established screening paradigms.

## Author Contributions

**Penghai Sun:** writing – original draft, investigation, conceptualization. **Zhenkai Fu:** writing – original draft, methodology, conceptualization. **Shaodong Wang:** writing – review and editing, software, methodology. **Yun Li:** writing – review and editing, conceptualization, supervision, resources. **Mantang Qiu:** writing – review and editing, conceptualization.

## Funding

This work was supported by the Science and Technology Innovation Program of Xiongan New Area, 2023XAGG0071; National Key R&D Program of China, 2023YFF0723500; Beijing Nova Program, 20230484314.

## Conflicts of Interest

The authors declare no conflicts of interest.

## Data Availability

Data sharing is not applicable to this article as no new data were created or analyzed in this study.
